# Early results of fixed-bearing unicompartmental knee replacement designed for the lateral compartment

**DOI:** 10.1186/s13018-021-02896-3

**Published:** 2022-03-05

**Authors:** Saeed Asadollahi, Hannah A. Wilson, Fraser R. Thomson, Kenneth Vaz, Rob Middleton, Cathy Jenkins, Abtin Alvand, Nicholas Bottomley, Chris A. Dodd, Andrew J. Price, David W. Murray, William F. Jackson

**Affiliations:** 1 Nuffield Orthopaedic Centre, Oxford University Hospitals NHS Foundation Trust, Windmill Road, Headington, Oxford, OX3 7LD UK; 2 Nuffield Department of Orthopaedics, Rheumatology and Musculoskeletal Sciences, Botnar Research Centre, University of Oxford, Oxford, UK

**Keywords:** Arthroplasty, Replacement, Knee, Knee prosthesis

## Abstract

**Background:**

Isolated lateral compartment knee arthritis is less prevalent than medial. While the reported results of medial unicompartmental knee replacement (UKR) have been good and comparable to total knee replacement, the results of lateral UKR have been mixed. We present the short-term results and survivorship of a fixed-bearing UKR designed specifically for the lateral compartment.

**Methods:**

We report the result of 130 primary fixed-bearing lateral Oxford (FLO) UKRs (123 patients) performed between 2015 and 2019 with a minimum follow-up of 1 year. The indications for lateral UKR were: isolated lateral osteoarthritis (*n* = 122), post-trauma (*n* = 5) and osteonecrosis (*n* = 3). The mean age was 69.1 (± 11.6), mean BMI 28.4 (± 4.9), 66.9% female, 60% right-sided, and mean follow-up 3 (range 1–4.8 years, standard deviation ± 1) years. The primary outcome measure was the Oxford knee score (OKS). Survival analysis was performed with “revision for any reason”, “reoperation”, and “implant failure” as the endpoints.

**Results:**

Six patients died from unrelated reasons. None of the implants failed. One required the addition of a medial UKR for medial arthritis. There were no other reoperations. At 4 years, the survival for implant failure was 100% and for both revision and all reoperations was 99.5% (95% CI 96.7–99.9%). At the last review, at a mean of 3 years, the mean Oxford knee score was 41.

**Conclusion:**

The good survivorship and outcome scores suggest that UKR designed for the lateral compartment is an excellent alternative to total knee replacement in selected patients with isolated lateral tibiofemoral arthritis at short-term follow-up.

## Introduction

Isolated lateral compartment osteoarthritis affects approximately 10% of patients with knee osteoarthritis [1]. If these patients present with symptoms and damage suitable for joint replacement, they can be managed with a total knee arthroplasty or a lateral UKR [2–4].

A UKR has potential advantages over total knee replacement. It is less invasive, preserves bone stock, and leaves the unaffected contralateral tibiofemoral compartment intact [5]. The procedure is associated with a faster recovery, a better ROM, less complication, shorter length of stay, higher likelihood of forgetting the artificial joint, and better cost-effectiveness [3, 4, 6–11]. Many surgeons, however, choose to use total knee replacements as they fear high revision rates of UKR seen in Joint registries around the globe [12–15]. They are also anxious because of mixed outcomes that have been previously reported [16–20].

There are two philosophies in use with UKR, mobile and fixed bearings. Mobile bearings have the advantage of a lower rate of polyethylene wear and improved kinematics [21]. The main drawback with this design is bearing dislocation [22–25]. This is more problematic in the lateral compartment, which distracts by about 7 mm in flexion compared to 2 mm on the medial side [26]. The Oxford domed lateral UKR was developed featuring an entirely congruous articulation using a biconcave spherical bearing which increased entrapment and therefore reduced the overall dislocation rate but did not eliminate it [27].

The FLO partial knee replacement (Zimmer Biomet UK, Bridgend) was designed to achieve optimal coverage of the lateral tibia plateau based on more than 400 knee CT scans [28]. The flat articulation allows soft tissues to guide the femoral component during flexion and extension [28]. As with other fixed-bearing designs, the kinematics, especially as the knee goes into higher degrees of flexion are not as optimal as with the domed design. The FLO and domed lateral were designed to be interchangeable, allowing the surgeon to choose intra-operatively whether to implant mobile or fixed-bearing device after assessing bearing stability at the final trial.

To date, there are only few published reports on the clinical outcome and survivorship of fixed-bearing lateral UKR with large variability in clinical results and longevity [17, 18, 29–35]. The primary objective of this study was to assess the clinical effectiveness of the FLO partial knee replacement in a large cohort of patients. Our secondary objectives were to investigate complications and survivorship of this new implant in the short term.

## Materials and methods

This study has been performed in accordance with the ethical standards laid down in the 1964 Declaration of Helsinki and its later amendments. This is a retrospective review of prospectively collected data on patients who underwent FLO lateral unicompartmental knee replacement. We identified 141 knees in 134 patients who underwent primary FLO lateral unicompartmental knee replacement between 2015 and 2019 and were followed for a minimum of 1 year. Bi-compartmental UKRs were excluded. Two patients were lost to follow-up. Three patients who had ACL deficiency at the time of surgery were also excluded. Six patients died for unrelated reasons at a mean of 2.1 years post-surgery. The remaining 123 patients (130 UKRs) were included and are the focus of this study.

All procedures were performed by the orthopaedic consultants who routinely perform UKR in our centre. The indications for lateral UKRs were: bone-on-bone disease in the lateral compartment or spontaneous osteonecrosis of the knee in the lateral compartment, full-thickness cartilage in the medial compartment, intact collateral and anterior cruciate ligaments and a correctable intra-articular deformity. Inflammatory arthritis and fixed valgus deformity were considered to be a contraindication. Patient factors such as age, weight, activity level, and patellofemoral joint damage proposed by Kozinn and Scott in 1989 to be contraindications for UKR have not been considered to be contraindications for the fixed-bearing UKR [36, 37]. Severe wear of the lateral facet of the patellofemoral joint with bone loss and grooving is also a contraindication for UKR [38].

All procedures were performed under a tourniquet through a small lateral para-patellar approach without dislocation of the patella. A trans-patellar tendon vertical tibial cut was performed to facilitate orientation of the saw cut and internal rotation of the tibial component. The femoral component was anatomically positioned to avoid an elevation of the joint line. The bearing thickness was assessed with the knee in full extension. All tibial components were secured with polymethylmethacrylate cement. Full weight-bearing was allowed postoperatively.

The electronic patient records were reviewed for demographics, details of the operation report, BMI, and any complications during follow-up assessment visits.

Survivorship analysis was performed with the endpoint “failure of implant”, “revision for any reason” and “reoperation”. “Revision for any reason” was defined as operation in which at least one component was removed or changed, or a new component was added. The outcome was assessed with the latest OKS [39].The OKS was categorised into excellent (> 41), good (34–41), fair (27–33), poor (< 27) [40].

### Statistical analysis

Statistical analysis was performed using the SPSS version 20.0 software program (SPSS, Chicago, IL). Simple descriptive and frequency analysis was performed on multiple variables. Shapiro–Wilk and Kolmogorov–Smirnov tests were used to test for normal distribution. Unpaired T test was used to compare the means of normally distributed variables. The Mann–Whitney U test was used to compare the means for numeric data when the data distribution was not normal. JMP 15.1.0 statistical software (SAS, Cry, NC, USA) was used for Kaplan–Meier survival analysis and 95% confidence interval (CI) calculations. A p-level of less than 0.05 was considered statistically significant.

## Results

The mean follow-up was 3.0 years (range 1–4.8 years, SD ± 1). The right side was operated on in 60% of cases. The mean age was 69.1 (± 11.6) and the mean BMI 28.4 kg/m^2^ (± 4.9 kg/m^2^) (Table 1). There were no intra-operative complications, implant or wound infections or thromboembolic events in our cohort. None of the patients required a blood transfusion.

**Table 1:** Patients’ characteristics

Demographics	
Patients (knees)	123 (130)
Male: female	43:87
Mean age (years) (range)	69.1 (37–91)
Male	68.6 (38–85)
Female	69.1 (37–91)
Mean body mass index (kg/m^2^) (range)	28.4 (20.8–40.6)
Male	28 (21.2–33.6)
Female	28.6 (20.8–40.6)
Indication for lateral UKR	
Bone on bone lateral compartment OA	*n* = 122
Post-trauma arthritis	*n* = 5
Osteonecrosis of the lateral femoral condyle	*n* = 3

There was one recorded postoperative acute kidney injury. This was appropriately medically managed without further complications. One knee required an intra-articular injection of steroid and local anaesthetic 18 months postoperatively due to ongoing lateral sided knee pain, after which the symptoms settled.

Outcome scores were obtained for 121 knees (93%). The mean preoperative OKS was 21.3 (SD 8.44, *n* = 76). The OKS improved significantly (*p* < 0.001) following the operation. At a mean of 3 years postoperatively, the mean OKS was 41 (SD 7.6, *n* = 121). Sixty per cent achieved an excellent outcome, 29% a good outcome, 5% a fair outcome, and 6% a poor outcome. The causes for the fair and poor results were persistent or lingering pain in the knee, pain in the contralateral leg, or concurrent medical condition (polyneuropathy, acute myeloid leukaemia) interfering with their activities of daily living.

The preoperative OKS was not available for 54 knees. However, this group mainly had similar demographics to the preoperative group, including age (69 vs 70 years old, *P* = 0.6), BMI (29 versus 27 kg/m^2^, *p* = 0.62), and the operative side (*p* = 0.43). There were more female patients in the group with unavailable preoperative OKS (*p* = 0.051).

A sensitivity analysis for selection bias and unmeasured confounding in missing data was performed, including a subgroup analysis of only those with recorded preoperative OKS. The result of the subgroup analysis demonstrated a statistically significant increase in the OKS (21.5 versus 39.5, *p* < 0.001).

**Fig. 1:**
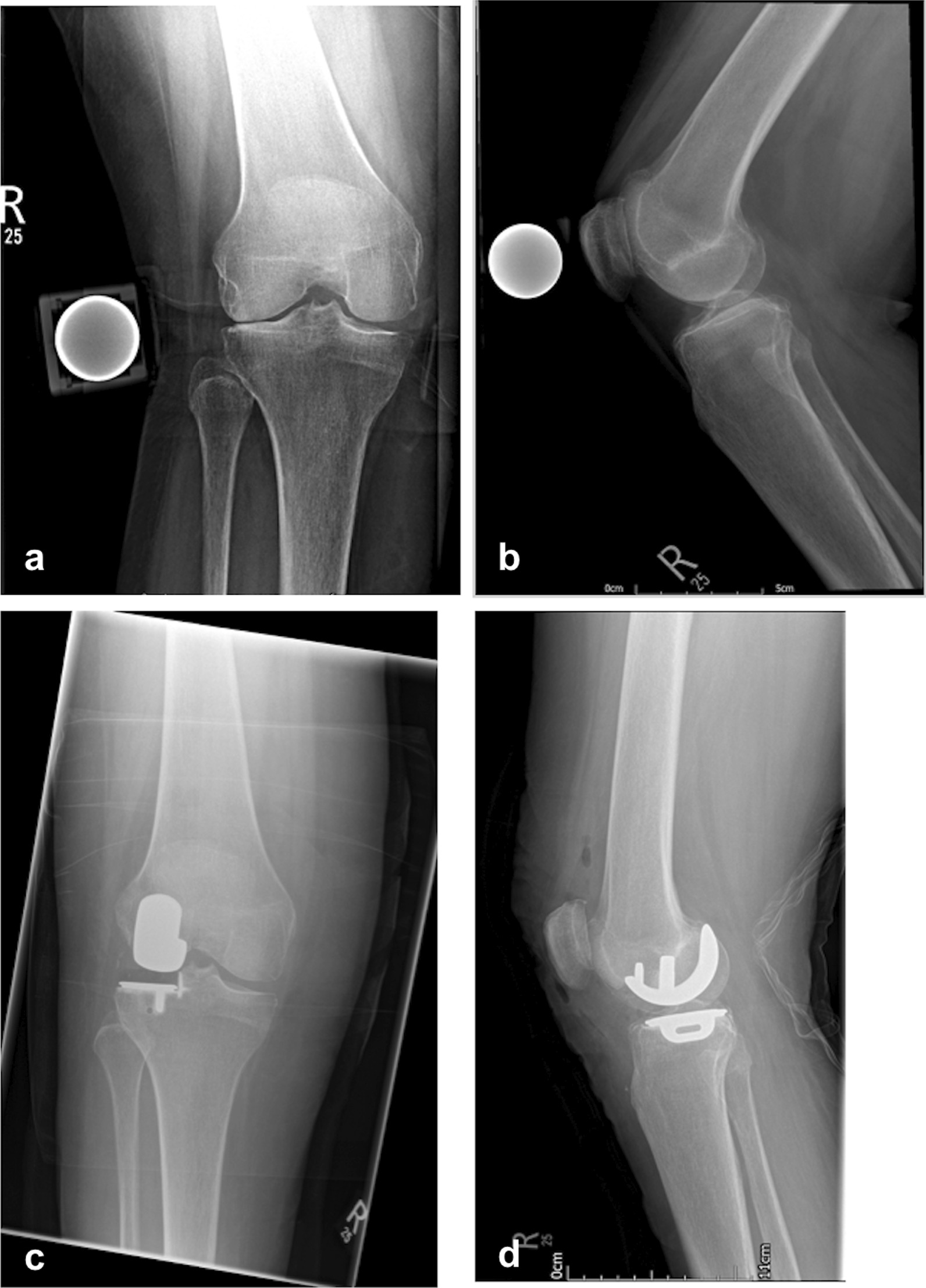
**a**–**d** Pre- and postoperative X-ray of the right knee with isolated lateral OA which underwent L UKR

There was one reoperation in this series, which was the addition of a medial UKR at 1 year for progression of medial compartment arthritis in a 70-year-old male. This patient was keen to have a Lateral UKR for severe lateral OA, even though he had some early medial OA. Initially, he did well following the FLO; however, he developed medial symptoms associated with the progression of medial OA. This was treated with a Medial UKR, and he had a good result from the Bi-UKR. His final OKS score was 45 (Fig. 1).

**Fig. 2:**
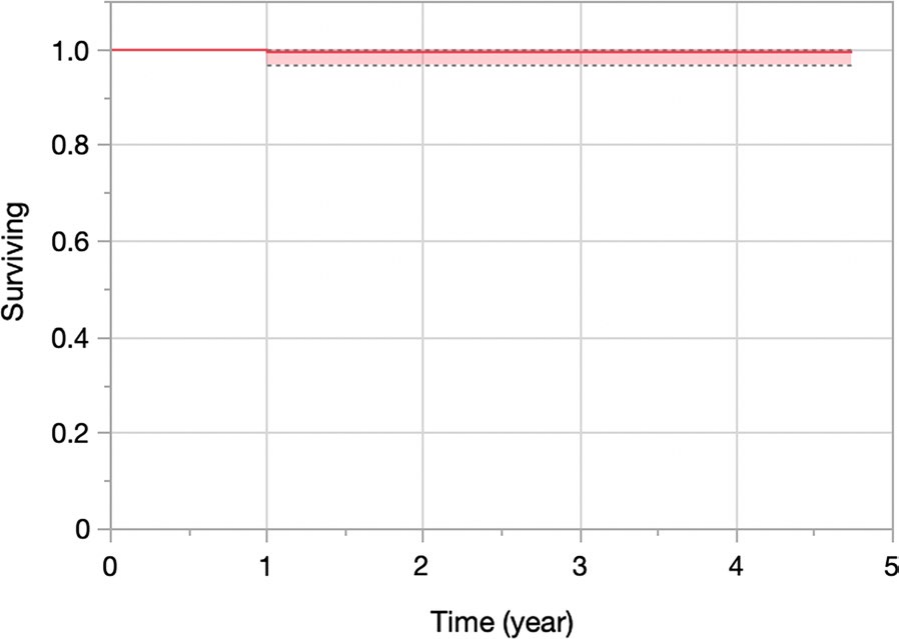
Kaplan–Meier survival curve of FLO partial knee replacement with the endpoint “revision for any reason”

**Fig. 3:**
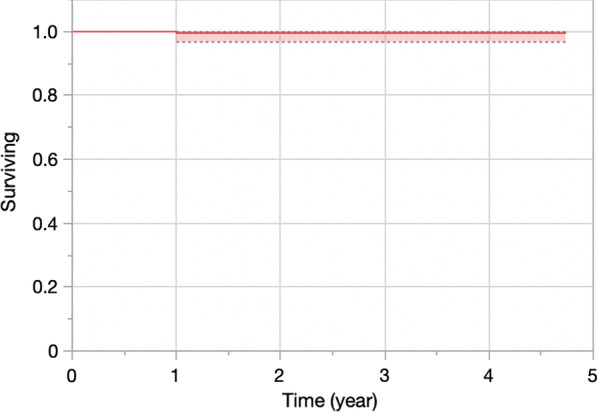
Kaplan–Meier survival curve of FLO partial knee replacement with the endpoint “any reoperation”

Kaplan–Meier survival analysis at 4 years with the endpoints “revision for any reason” and “any reoperation” was 99.5% (95% confidence interval (CI) 96.7%–99.9%) (Figs. 2, 3). There were no implant failures, so the 4-year survival for implant failure was 100%.

## Discussion

This short-term study has demonstrated that patients tended to achieve a satisfactory functional outcome with a low complication rate when treated with a FLO UKR, suggesting that it is a viable option for patients with isolated lateral tibiofemoral osteoarthritis.

We demonstrated implant survivorship of 99.5% at 4 years in 130 knees with endpoint “revision for any reason”. Several other series have reported the survivorship of fixed-bearing lateral UKR, with some demonstrating similarly good results: Recently, Walker et al. reported a FLO partial knee replacement survival rate of 100% at 2 years in a series of 51 patients [29]. Smith et al. showed survivorship of 95.5% at 5 years in a series of 101 AMC Uniglide implants (Corin PLC, Cirencester, UK) [33]. Edmiston et al. reported survivorship of 94% at a mean of 82 months in a series of 49 Zimmer unicompartmental knee (Zimmer, Warsaw, IN) or Zimmer Miller–Galante implants. Similar to the current study, there was no nonrevision reoperation reported in these series.

Furthermore, the survivorship result of the current study compares favourably to registry data. The data analysis from the National Joint Registry for England and Wales (NJR) shows a 93.0% survival rate for lateral UKRs at 5 years [41]. A recently published report of Dutch registry data shows 8.9% and 12.9% revision rates at 3 and 5 years, respectively [15]. This variation in survival rate highlights the ongoing discrepancy between outcomes of UKR from cohort series and registries [8]. The discrepancy probably relates to the surgeon’s practice. Most surgeons who report good results from cohort studies tend to be implanting reasonable numbers of UKR, whereas the commonest number of UKR done per surgeon per year recorded by the NJR is one [42]. Furthermore, often these surgeons use UKR inappropriately for patients with early arthritis [43].

The present study found that the mean 3-year postoperative OKS was 41, which is considered to be excellent and similar to the medial Oxford UKR (mean OKS 42 at 5 years) [44]. However, the improvement compared to preoperative score tended to be larger following lateral UKR (mean ΔOKS 20) than medial UKR (meanΔOKS 17). This is probably a reflection of the lower preoperative score of the Lateral UKR (mean OKS 21) than the medial (mean OKS 25). This, in turn, is because many of the patients we treat with lateral UKR have more severe disease with greater deformity than would be appropriate for medial UKR. Interestingly in Walker et al. report, the mean preoperative FLO OKS was 26.4, ΔOKS was 13.3, and 2 years OKS was 39.7, perhaps suggesting they were operating on less severe lateral disease than we do [29].

Progression of disease in the adjacent compartments is the most common reason for early and late failure following lateral UKR [19, 23, 27, 45]. One of the problems lies in overcorrection after UKR [19]. A few studies with varying follow-up durations reported a revision rate of 0 to 10.2% due to contralateral OA progression 1.1 to 18.1 years after the initial surgery [16, 18, 19, 25, 33, 46]. In the current series, there was one instance of the addition of a medial UKR at 1 year due to OA progression. This was probably a manifestation of an extended indication at the request of a high demand individual wanting to maintain a high-level activity. In order to avoid overcorrection of the deformity, we implant the femoral component anatomically and do not balance the ligaments so in flexion the normal laxity of the knee is maintained. The bearing thickness is selected in full extension, with the aim being just not to tighten the knee, so the pre-disease valgus is restored or slightly undercorrected.

This study has some limitations. The follow-up is short, making it impossible to draw any conclusion about the long-term outcome of the FLO partial knee replacement. There is a lack of a control group to compare this implant to other available fixed-bearing implants in the market. Nearly 40% of patients did not have a preoperative OKS recorded. However, there was no significant statistical difference in demographics between this group and the rest of the cohort, except for more female patients (*p* = 0.051). Furthermore,  the sensitivity analysis showed that the mean postoperative OKS of those with recorded pre-operative OKS was similar to those without a recorded pre-operative OKS.

To our knowledge, this study is the largest series of the FLO partial knee replacement reported in the literature. These results show outcomes that have been observed in a high-volume centre with a specialist interest in UKR surgery. Additional work needs to be done to ensure these results are transferable to the general orthopaedic community. Further follow-up is also necessary to evaluate the mid and long-term effectiveness of the FLO partial knee replacement.

## Conclusions

The early results show good functional outcome and implant survival in the knees treated with the FLO partial knee replacement. It is an attractive option for the management of isolated osteoarthritis of the lateral compartment.

## Data Availability

Data are available on request.

## Abbreviations

UKR: Unicompartmental knee replacement
ROM: Range of motion
FLO: Fixed-bearing lateral Oxford
BMI: Body mass index
OKS: Oxford knee score
OA: Osteoarthritis
